# Physiological Responses in the Hepatopancreas of *Litopenaeus vannamei* to Carbonate Alkalinity Stress and Subsequent Recovery: Integration of Antioxidant, Immune, and Metabolic Profiles

**DOI:** 10.3390/antiox15091052

**Published:** 2026-08-23

**Authors:** Ruijie Zhu, Meng Xiao, Falin Zhou, Zhe Pan, Jianhua Huang, Yafei Duan

**Affiliations:** 1 State Key Laboratory of Mariculture Biobreeding and Sustainable Goods, Key Laboratory of South China Sea Fishery Resources Exploitation & Utilization, Ministry of Agriculture and Rural Affairs, South China Sea Fisheries Research Institute, Chinese Academy of Fishery Sciences, Guangzhou 510300, China; zhurj1014@163.com (R.Z.); xiaomeng20240415@163.com (M.X.); zhoufalin@scsfri.ac.cn (F.Z.); huangjianhua@scsfri.ac.cn (J.H.); 2 Ocean College, Hebei Agricultural University, Qinhuangdao 066003, China; lfpanzhe@163.com; 3 Key Laboratory of Efficient Utilization and Processing of Marine Fishery Resources of Hainan Province, Sanya Tropical Fisheries Research Institute, Sanya 572018, China; 4 Southern Marine Science and Engineering Guangdong Laboratory (Zhuhai), Zhuhai 519080, China; 5 Shenzhen Base of South China Sea Fisheries Research Institute, Chinese Academy of Fishery Sciences, Shenzhen 518121, China

**Keywords:** shrimp, carbonate alkalinity, hepatopancreas, physiological function, metabolism

## Abstract

High carbonate alkalinity (CA) is a major constraint on shrimp culture in saline–alkaline waters. The hepatopancreas is central to shrimp immunity and metabolic regulation. *Litopenaeus vannamei* underwent a 7-day exposure period to 5 mmol/L CA, followed by a subsequent 7-day recovery phase after removal of the stressor. The physiological regulatory mechanism of the hepatopancreas during CA stress and recovery was investigated by integrating multiple biological levels including histomorphology, antioxidant and immune indices, energy metabolism, and metabolite profiles. Results showed that CA stress induced structural changes in the hepatopancreas and triggered stress responses. Specifically, a significant upregulation was observed in genes involved in antioxidation (*romo1*, *nrf2*, *gpx*, *hsp70*), apoptosis (*casp-9*, *casp-3*), endoplasmic reticulum (ER) stress (*ire1*, *xbp1*), immune defense (*alf*, *crus*, *pen-3*, *lys*, *propo*), and detoxification (*cyp450*). CA stress also increased osmoregulatory genes (*ccp*, *nhe*, *ca*, *aqp*, *vatp*, *nka-β*, *nka-α*), whereas *clc* and *tip4* were suppressed. CA stress reduced the levels of energy-metabolism-related biochemical indicators, including glucose (GLU), pyruvic acid (PYR), lactic acid (LAC) and triglycerides (TG), while markedly inducing the expression of genes involved in carbohydrate metabolism (*ldh*, *pdh*, *hk*, *pk*), lipid metabolism (*ampk*, *srebp*, *fas*), the tricarboxylic acid (TCA) cycle (*mdh*, *cs*, *idh*, *odh*, *sdh*, *fh*), and the electron transport chain (ETC) (*ndh*, *cytc*, *coi*, *cco*, *atph*). Moreover, the hepatopancreatic metabolic profile was remodeled, especially “phenylalanine, tyrosine and tryptophan biosynthesis” and the metabolism of *β*-alanine, arachidonic acid, linoleic acid, and sphingolipids being substantially altered during both the stress and recovery phases. Several functional metabolites linked to stress responses were further pinpointed. Following stress relief, some physiological parameters partially recovered, yet overall function failed to return to normal. Collectively, CA stress compromised hepatopancreatic homeostasis by damaging morphological integrity, eliciting stress and immune responses, and perturbing energy metabolism and metabolite homeostasis; these adverse effects were not readily reversible in the short term.

## 1. Introduction

*Litopenaeus vannamei* is an important cultured shrimp, attributed to its rapid growth rate, robust tolerance to stress, and adaptability to high salinity conditions. Moreover, it shows considerable potential for aquaculture in saline-alkaline waters [1]. Saline-alkaline waters are extensively distributed worldwide, covering an area of 950 million hectares [2]. Yet, due to their elevated pH, substantial alkalinity, and complicated ionic makeup, saline-alkaline waters are largely uninhabitable for the majority of aquatic organisms [3]. Carbonate alkalinity (CA) represents the acid-neutralizing capacity contributed primarily by bicarbonate (HCO_3_^−^) and carbonate (CO_3_^2−^) in water [4]. Although these carbonate species participate in normal physiological processes, excessive CA may disturb acid–base and ionic gradients between aquatic organisms and the surrounding water, thereby interfering with ion transport, osmoregulation, and physiological homeostasis [3,5]. Previous studies have reported that a CA level of 2.90 mmol/L was considered tolerable for *L. vannamei* larvae reared at low salinity (3.29–4.84‰) [6]. However, in saline-alkaline aquaculture ponds, CA concentrations ranging from 1.4 to 8.0 mmol/L and from 3.5 to 13.0 mmol/L have been reported under different environmental conditions [7]. Elevated CA levels can disrupt the normal physiological functions of shrimp and affect their survival and growth [5,8,9]. Consequently, investigating the physiological mechanisms by which shrimp respond to CA stress will aid in formulating effective anti-stress strategies.

The hepatopancreas functions as the primary organ for metabolism and detoxification in shrimp [1]. CA stress impairs the physiological functions of the shrimp hepatopancreas through multiple mechanisms. For instance, CA stress can drive hepatopancreatic overproduction of reactive oxygen species (ROS) in *L. vannamei*, disturb antioxidant enzyme homeostasis, and ultimately trigger oxidative stress [10]. Moreover, CA stress modulates immunity gene expression and modifies the hepatopancreatic physiological regulatory networks of *L. vannamei* [5]. Apoptotic and detoxification signaling pathways are also altered in the hepatopancreas of *L. vannamei* under CA stress [5,11]. Comparable oxidative injury, immune impairment, and inflammation have also been observed in the hepatopancreas of *Fenneropenaeus chinensis* [12].

CA stress also influences the metabolic balance within the hepatopancreas of shrimp. In the hepatopancreas of *L. vannamei*, reported metabolic alterations have been associated with diverse metabolic processes, encompassing nitrogen metabolism, nutrient transport and utilization, carbohydrate and lipid metabolism, as well as amino acid and nucleotide metabolism [5,9,10,13]. In addition, CA stress can also activate adenosine 5′-monophosphate (AMP)-activated protein kinase (AMPK) and insulin signal pathways in the hepatopancreas of *Macrobrachium nipponense*, causing core functional disorders such as energy metabolism and lipid metabolism, accompanied by abnormal immune response [14].

Previous studies have described the physiological, molecular, and metabolic responses of *L. vannamei* to CA stress. However, most have examined individual physiological parameters or specific metabolic pathways during the stress period. Relatively little attention has been paid to the dynamic responses occurring across both the stress and recovery phases or to the coordinated regulation of multiple physiological functions. Accordingly, the present study investigated the hepatopancreatic responses of *L. vannamei* during CA stress and subsequent recovery. We hypothesized that CA stress would cause hepatopancreatic tissue damage and disrupt cellular stress responses, immune function, osmoregulation, energy metabolism, and metabolic profiles. These alterations were expected to recover to varying degrees after the removal of CA stress. To evaluate this hypothesis, *L. vannamei* reared under low-salinity conditions underwent CA treatment at 5 mmol/L for one week, after which the animals were allowed to recover for an additional week following stress removal. The effects of CA stress on the hepatopancreas were investigated through analyses of morphology, oxidative and endoplasmic reticulum (ER) stress responses, immune-related changes, apoptotic regulation, detoxification, osmoregulation, and energy metabolism. Metabolomic profiling was further used to identify pathways and functional metabolites associated with CA stress and recovery. These analyses will contribute to understanding the physiological regulatory mechanisms of the shrimp hepatopancreas in response to CA stress and will aid in the development of appropriate anti-stress strategies.

## 2. Materials and Methods

### 2.1. Shrimp and Culture Conditions

We collected *L. vannamei* (9.6 ± 0.4 g) from an indoor aquaculture facility at the Shenzhen Base of the South China Sea Fisheries Research Institute (Shenzhen, China). Before exposure, the shrimp underwent a 7-day acclimation phase in 300 L tanks maintained at 25 ± 0.5 °C, salinity 3‰, and pH 8.2 ± 0.2, with continuous aeration and complete daily water exchange. They were then fed a formulated diet at 5% of body weight, with feeding rates adjusted according to consumption. To ensure stable water conditions, waste and uneaten feed were removed promptly.

### 2.2. CA Stress Test and Sample Collection

One week of acclimation was followed by random assignment of shrimp to a CA stress group or a control group (CK). Each group was established with three replicate tanks, each having an inner diameter of 98.0 cm, a depth of 100.0 cm, and an effective water volume of 300 L. A total of 50 shrimp were stocked in each tank. Considering the CA levels reported in saline-alkaline aquaculture ponds and previous studies [5,6,7,9], the present study employed 5 mmol/L CA as the stress concentration. Shrimp in CK were reared in 3‰ low-salinity water, while the CA group was exposed to 5 mmol/L CA by adding sodium bicarbonate. A 100% water exchange was performed daily in each tank using pre-prepared experimental water, and other culture conditions remained unchanged. CA was determined by acid–base titration. According to previous studies, a 7-day stress duration was adopted to investigate the response of *L. vannamei* to CA stress. A 7-day recovery period has also been shown to reflect short-term physiological recovery patterns following stress removal [6,15,16]. To establish a recovery group (RCA), the CA-treated water was replaced with standard 3‰ low-salinity water following the initial CA stress phase. Subsequently, 15 shrimp from each CA tank were transferred to normal culture conditions for a further 7 days.

Hepatopancreas samples were collected immediately after the CA treatment phase and after completion of the subsequent recovery phase. Six shrimp were randomly sampled from each tank, and their hepatopancreas were divided into two halves: one half was used for biochemical analysis, and the other half for metabolomic analysis. Another three shrimp specimens were collected and preserved in RNA stabilizer for subsequent quantitative real-time polymerase chain reaction (qPCR) analysis, whereas another three were fixed in 4% paraformaldehyde for histological examination.

### 2.3. Histomorphological Analysis

Hepatopancreatic tissues were fixed in 4% paraformaldehyde for 24 h and subsequently dehydrated through an ethanol series, cleared with xylene, and infiltrated with paraffin. The prepared sections (4 μm thick) were subjected to haematoxylin–eosin staining and examined microscopically for histological alterations.

### 2.4. Biochemical Indexes Determination

Frozen hepatopancreas samples were thawed, rinsed with normal saline, blotted with filter paper, and weighed. The tissue was then homogenized at low temperature using a tissue homogenizer (TissueLyser II, Qiagen, Hilden, Germany) with a mass ratio of tissue to normal saline of 1:9. We centrifuged the homogenate at 4 °C for 15 min at 1370× *g* (Centrifuge JIDI-21R, Guangzhou, China), collected the supernatant, and stored it at −80 °C for future biochemical measurements. Using commercial kits and standard methods, we then quantified glucose (GLU), pyruvic acid (PYR), lactic acid (LAC), triglycerides (TG), and adenosine triphosphate (ATP).

### 2.5. Gene Expression Analysis

Total RNA was isolated from the hepatopancreas with TRIzol reagent, followed by DNase treatment to remove residual genomic DNA. RNA quality was then assessed on the basis of concentration, purity, and integrity. cDNA was synthesized from the purified RNA through a reverse transcription reaction. The National Center for Biotechnology Information (NCBI) database was used to obtain target gene sequences and the *β-actin* reference sequence. Primer Premier 5.0 was employed for primer design, and the specificity of the primers was subsequently verified (Table S1). qPCR was carried out to detect gene expression using a standard reaction mixture and cycling conditions. Relative gene expression was calculated based on Livak and Schmittgen [17] and normalized to the CK group.

### 2.6. Non-Targeted Metabolomics Analysis

After thawing at 4 °C, metabolites were extracted from the hepatopancreas samples, with 20 µL from each sample reserved for quality control (QC), while the remaining extracts were analyzed by liquid chromatography–tandem mass spectrometry (LC–MS/MS; Thermo Vanquish system and Orbitrap Exploris 120 mass spectrometer, Thermo Fisher Scientific, Waltham, MA, USA). Raw data were converted and processed via Proteowizard and XCMS in R, followed by QC/quality assurance (QA) and auto-scaling standardization. Multivariate analysis using partial least squares discriminant analysis (PLS-DA) was performed, and differential metabolites between groups (CA vs. CK and RCA vs. CA) were screened based on *p* < 0.05 and variable importance in projection (VIP) > 1. The false discovery rate (FDR) was controlled using the Benjamini–Hochberg (BH) procedure. Metabolites were identified using Metlin, MoNA and a self-built database, classified using Kyoto Encyclopedia of Genes and Genomes (KEGG), and clustering analysis was conducted via pheatmap in R (v1.0.12). KEGG pathway analysis and metabolic network construction were performed, with further analysis on functional metabolites related to organism health.

### 2.7. Statistical Analysis

Values are reported as means ± standard error (SE). Statistical comparisons were conducted in SPSS 27.0 using one-way analysis of variance (ANOVA), followed by least significant difference (LSD) multiple range tests for post hoc analysis. A threshold of *p* < 0.05 was used to define statistical significance.

Additional methodological details are provided in the Supplementary Materials.

## 3. Results

### 3.1. The Alterations in the Hepatopancreas Histomorphology

**Figure 1:**
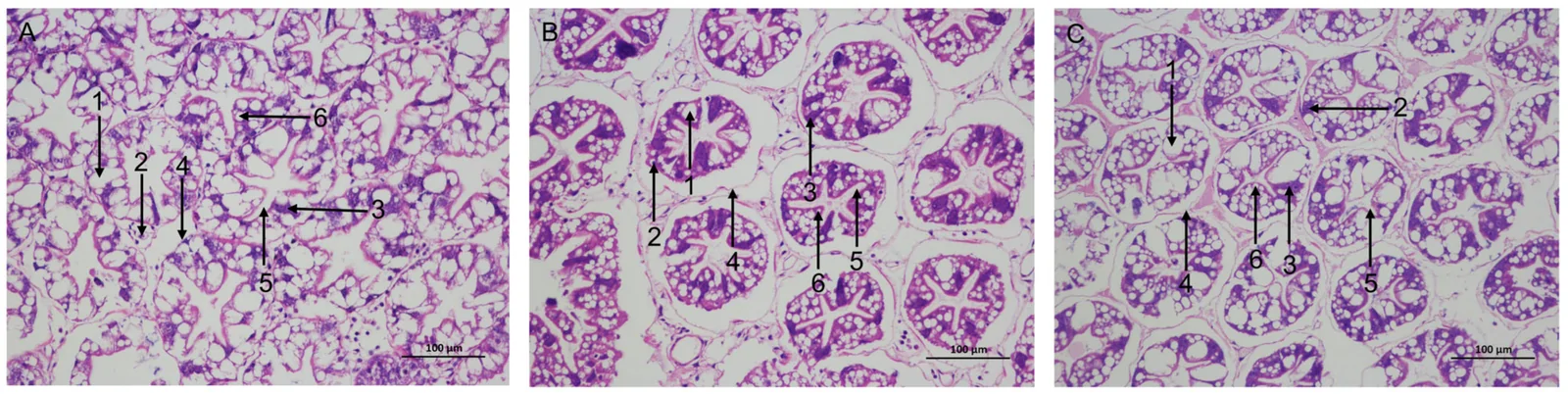
Histological changes in the hepatopancreas of *L. vannamei*. (**A**) The control (CK) group. (**B**) The carbonate alkalinity (CA) group. (**C**) The recovery (RCA) group. 1: secretory cells (B cells); 2: storage cells (R cells); 3: fibre cells; 4: basement membrane; 5: lumen; 6: transferred vacuoles. *n* = 3 tanks per group, with three shrimp per tank.

The CK group exhibited structurally intact hepatic tubules featuring star-shaped lumina. Uniform distribution of B cells was observed, and the intertubular connective tissues were closely associated with a clear basement membrane (Figure 1A). During the stress phase, hepatic tubule atrophy was noted in the CA group, characterized by separation from the basement membrane (Figure 1B). Following recovery, hepatic-tubule atrophy was less pronounced than after CA exposure (Figure 1C).

### 3.2. Changes in the Expression Levels of Antioxidant-Related Genes

**Figure 2:**
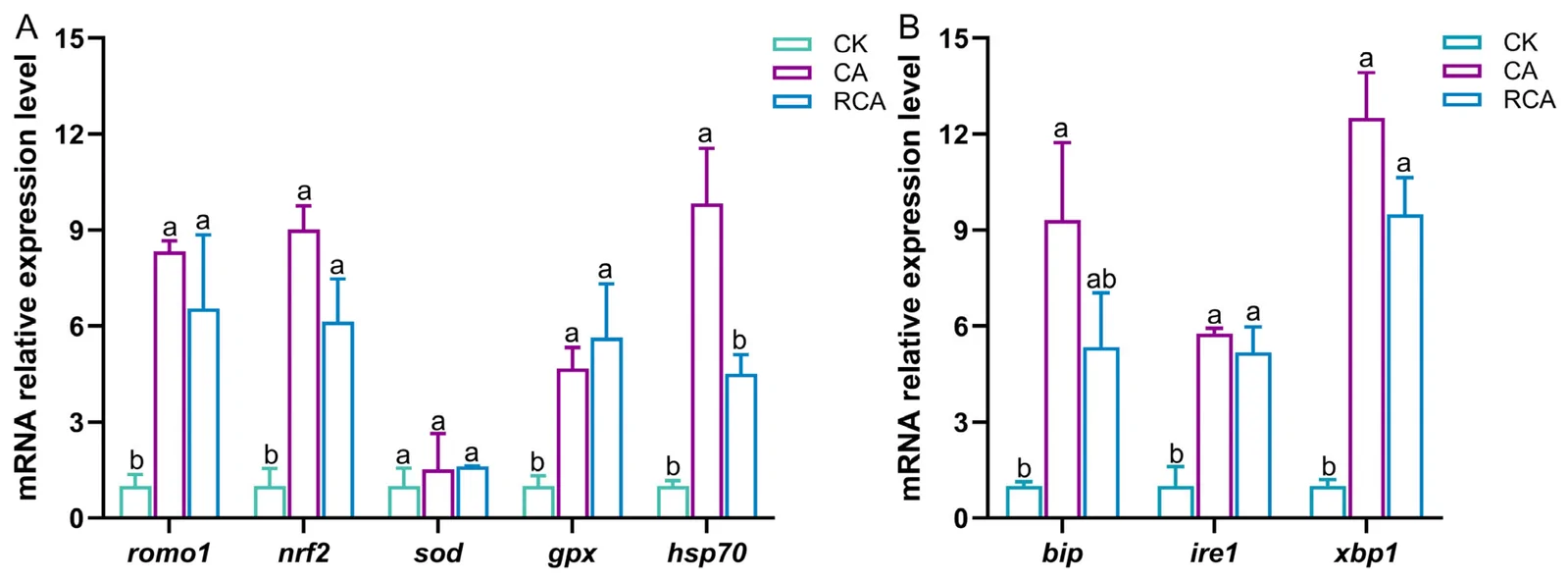
Expression of stress-response genes in the hepatopancreas during CA exposure and recovery. (**A**) Oxidative stress markers. (**B**) Endoplasmic reticulum (ER) stress markers. *n* = 3 tanks per group, with three shrimp sampled per tank. Groups labeled with different letters represent statistically significant differences (*p* < 0.05). Oxygen regulatory factor 1 (*romo1*); nuclear transcription factor 2 (*nrf2*); glutathione peroxidase (*gpx*); heat shock protein 70 (*hsp70*); superoxide dismutase (*sod*); immunoglobulin heavy chain binding protein (*bip*); inositol-requiring enzyme 1 (*ire1*); X-box binding protein 1 (*xbp1*).

Compared to the CK group, CA stress significantly increased oxygen regulatory factor 1 (*romo1*), nuclear transcription factor 2 (*nrf2*), glutathione peroxidase (*gpx*), and heat shock protein 70 (*hsp70*) expression (*p* < 0.05), whereas superoxide dismutase (*sod*) increased only slightly and not significantly (*p* > 0.05) (Figure 2A).

After recovery, the expression levels of *romo1*, *nrf2*, and *gpx* in the RCA group did not differ significantly from those in the CA group (*p* > 0.05), but remained significantly higher than those in the CK group (*p* < 0.05). In contrast, no significant variation in *sod* expression was detected between the RCA and CK groups (*p* > 0.05). Meanwhile, *hsp70* expression was reduced in the RCA group relative to the CA group (*p* < 0.05) and returned to a level comparable to that of the CK group (*p* > 0.05).

### 3.3. Changes in the Expression Levels of ER Stress-Related Genes

CA stress significantly elevated immunoglobulin heavy chain binding protein (*bip*), inositol-requiring enzyme 1 (*ire1*), and X-box binding protein 1 (*xbp1*) expression in the CA group relative to the CK group (*p* < 0.05) (Figure 2B).

After stress removal, the transcript levels of *bip*, *ire1* and *xbp1* showed a slight reduction in the RCA group relative to the CA group (*p* > 0.05). However, *ire1* and *xbp1* in the RCA group remained significantly higher than those in the CK group (*p* < 0.05), whereas *bip* transcription in the RCA group did not differ from the CK group (*p* > 0.05).

### 3.4. Changes in the Expression of Immune-Related Genes

**Figure 3:**
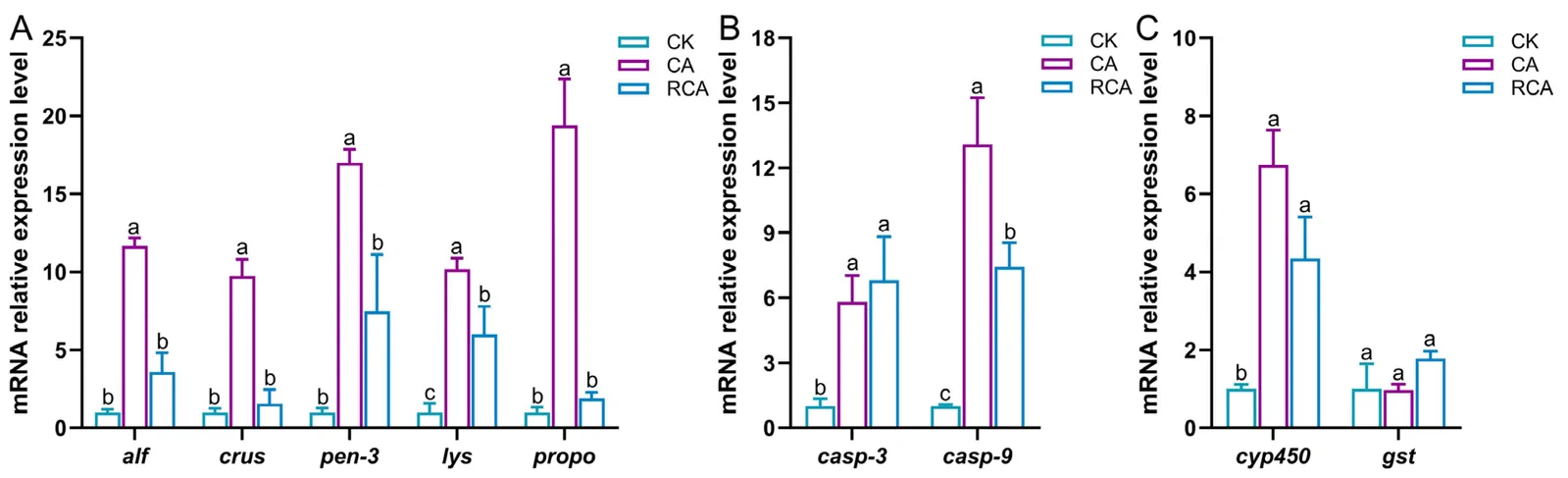
Hepatopancreatic expression profiles of genes related to immunity, apoptosis, and detoxification during CA stress and recovery. (**A**) Immunity-related genes. (**B**) Apoptosis-related genes. (**C**) Detoxification-related genes. *n* = 3 tanks per group, with three shrimp sampled per tank. Groups labeled with different letters represent statistically significant differences (*p* < 0.05). Anti-lipopolysaccharide factor (*alf*); crustin (*crus*); penaeidin 3 (*pen-3*); lysozyme (*lys*); phenoloxidase (*propo*); caspase-3 (*casp-3*); caspase-9 (*casp-9*); cytochrome P450 (*cyp450*); glutathione S-transferase (*gst*).

Relative to the CK group, CA stress significantly increased anti-lipopolysaccharide factor (*alf*), crustin (*crus*), penaeidin 3 (*pen-3*), lysozyme (*lys*), and phenoloxidase (*propo*) expression (*p* < 0.05) (Figure 3A).

During recovery, *alf*, *crus*, *pen-3*, and *propo* in the RCA group declined significantly from CA values (*p* < 0.05) and returned to CK levels; *lys* also decreased significantly from CA values, although it remained above the CK group (*p* < 0.05).

### 3.5. Changes in the Expression Levels of Apoptosis-Related Genes

Relative to the CK group, the CA group showed significant increases in caspase-3 (*casp-3*) and caspase-9 (*casp-9*) expression (*p* < 0.05) (Figure 3B).

After stress removal, *casp-3* expression in the RCA group showed no obvious change relative to the CA group (*p* > 0.05), whereas *casp-9* was significantly downregulated (*p* < 0.05); nevertheless, both genes remained significantly higher than those in the CK group (*p* < 0.05).

### 3.6. Changes in the Expression Levels of Detoxifying Metabolism-Related Genes

Compared with the CK group, CA exposure significantly increased cytochrome P450 (*cyp450*) expression (*p* < 0.05), while glutathione S-transferase (*gst*) gene expression exhibited no significant alteration (*p* > 0.05) (Figure 3C).

Following recovery, *cyp450* expression in the RCA group showed a nonsignificant decline from the CA group (*p* > 0.05) but remained above the CK group (*p* < 0.05). *gst* expression was slightly higher in the RCA group than in the CA group, without reaching statistical significance with the CK group (*p* > 0.05).

### 3.7. Changes in the Expression Levels of Osmoregulation-Related Genes

#### 3.7.1. Osmoregulation-Related Enzymes

**Figure 4:**
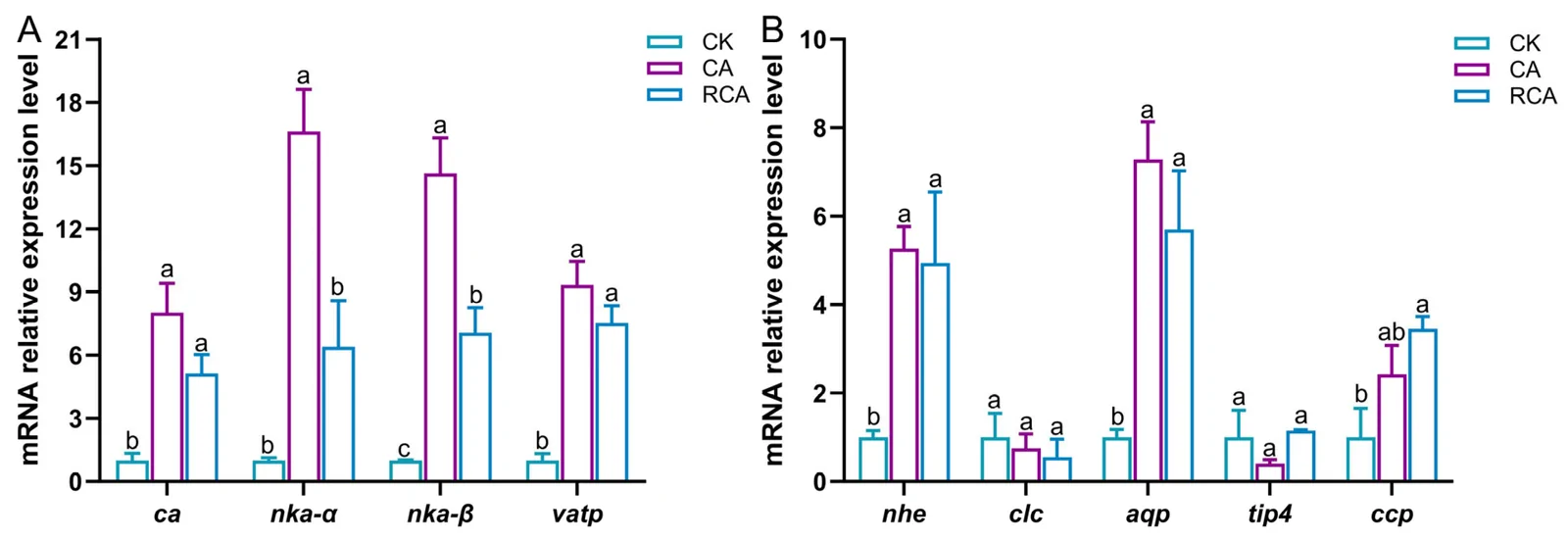
Osmoregulatory gene expression in the hepatopancreas during CA exposure and recovery. (**A**) Osmoregulation-related enzymes. (**B**) Osmoregulation-related proteins. *n* = 3 tanks per group, with three shrimp sampled per tank. Groups labeled with different letters represent statistically significant differences (*p* < 0.05). Carbonic anhydrase (*ca*); Na^+^/K^+^ ATPase α subunit (*nka-α*); Na^+^/K^+^ ATPase *β* subunit (*nka-β*); V-type proton ATPase subunit C (*vatp*); sodium/hydrogen exchanger (*nhe*); chloride channel protein 2 (*clc*); aquaporin (*aqp*); aquaporin tip4 (*tip4*); two-pore calcium channel protein 1 (*ccp*).

Compared with the CK group, carbonic anhydrase (*ca*), Na^+^/K^+^ ATPase α subunit (*nka-α*), Na^+^/K^+^ ATPase *β* subunit (*nka-β*), and V-type proton ATPase subunit C (*vatp*) showed significantly increased expression following CA stress (*p* < 0.05) (Figure 4A).

After recovery, *ca* and *vatp* expression in the RCA group showed a modest reduction from the CA group (*p* > 0.05), but remained higher than the CK baseline (*p* < 0.05). *nka-α* expression in the RCA group was significantly lower than that observed under CA stress (*p* < 0.05) and was comparable to the CK level (*p* > 0.05). By contrast, *nka-β* expression declined significantly compared with that in the CA group but remained elevated relative to the CK level (*p* < 0.05).

#### 3.7.2. Osmoregulation-Related Proteins

Compared with the CK group, CA stress significantly elevated sodium/hydrogen exchanger (*nhe*) and aquaporin (*aqp*) expression (*p* < 0.05). Chloride channel protein 2 (*clc*) and aquaporin tip4 (*tip4*) showed slight decreases, whereas two-pore calcium channel protein 1 (*ccp*) increased slightly; none of these three changes was significant (*p* > 0.05) (Figure 4B).

After recovery, *nhe* and *aqp* expression remained unchanged relative to the CA group (*p* > 0.05), although both maintained significantly elevated expression levels relative to the CK group (*p* < 0.05). There was no significant difference in *clc* expression between the RCA and CA groups, and its expression returned to the CK level (*p* > 0.05). A slight increase in *tip4* expression was observed in the RCA group, but it did not differ significantly from the CK group (*p* > 0.05). Compared with the CA group, *ccp* exhibited a mild elevation (*p* > 0.05), while its expression remained higher than the CK baseline (*p* < 0.05).

### 3.8. Changes in the Energy Metabolism-Related Indexes

#### 3.8.1. Glucose Metabolism

**Figure 5:**
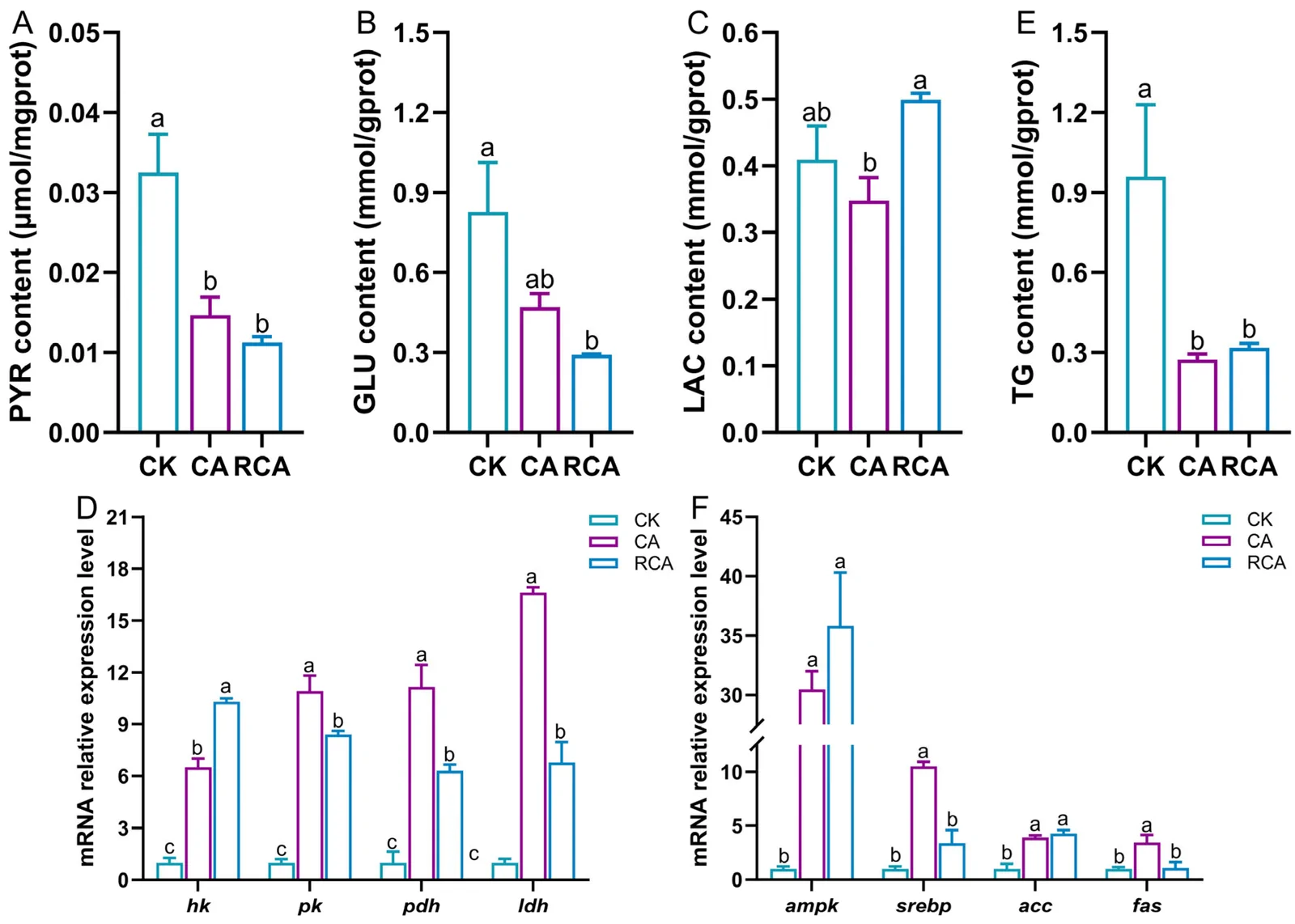
Hepatopancreatic glucose- and lipid-metabolism indices during CA stress and recovery. (**A**) Pyruvate (PYR) content. (**B**) Glucose (GLU) content. (**C**) Lactic acid (LAC) content. (**D**) Carbohydrate metabolism-related genes. (**E**) Triglyceride (TG) content. (**F**) Lipid metabolism-related genes. *n* = 3 tanks per group, with six shrimp sampled per tank for biochemical assays and three shrimp sampled per tank for gene expression analysis. Groups labeled with different letters represent statistically significant differences (*p* < 0.05). Acetyl-CoA carboxylase (*acc*); adenosine 5′-monophosphate (AMP)-activated protein kinase (*ampk*); cholesterol regulatory element binding protein (*srebp*); fatty acid synthase (*fas*); hexokinase (*hk*); lactate dehydrogenase (*ldh*); pyruvate dehydrogenase (*pdh*); pyruvate kinase (*pk*).

Following CA stress, PYR levels were significantly lower than those observed in the CK group (*p* < 0.05), whereas GLU and LAC exhibited only slight downward trends without statistical significance (*p* > 0.05) (Figure 5A–C). In addition, the expression of glycolysis-related genes, including hexokinase (*hk*), pyruvate kinase (*pk*), pyruvate dehydrogenase (*pdh*), and lactate dehydrogenase (*ldh*), was significantly higher in the CA group than in the CK group (*p* < 0.05) (Figure 5D).

Following recovery, GLU and PYR contents showed no significant alterations, although both exhibited a slight decreasing tendency relative to the CA group (*p* > 0.05), while both were significantly reduced compared with the CK group (*p* < 0.05). In contrast, LAC content exhibited a significant increase after recovery (*p* < 0.05) and showed no significant difference from the CK group (*p* > 0.05). The expression of *hk* remained significantly increased in the RCA group, whereas *pk*, *pdh*, and *ldh* expression was significantly decreased relative to the CA group, but all of them still exceeded CK levels (*p* < 0.05).

#### 3.8.2. Lipid Metabolism

CA stress significantly reduced TG content and increased *ampk*, cholesterol regulatory element binding protein (*srebp*), acetyl-CoA carboxylase (*acc*), and fatty acid synthase (*fas*) expression (*p* < 0.05) relative to the CK group (Figure 5E,F).

Following recovery, TG content, together with *ampk* and *acc* expression in the RCA group, remained stable relative to the CA group (*p* > 0.05), yet differed significantly from the CK group (*p* < 0.05); *srebp* and *fas* expression in the RCA group decreased significantly relative to the CA group (*p* < 0.05) and returned to CK levels.

#### 3.8.3. Tricarboxylic Acid Cycle (TCA)

**Figure 6:**
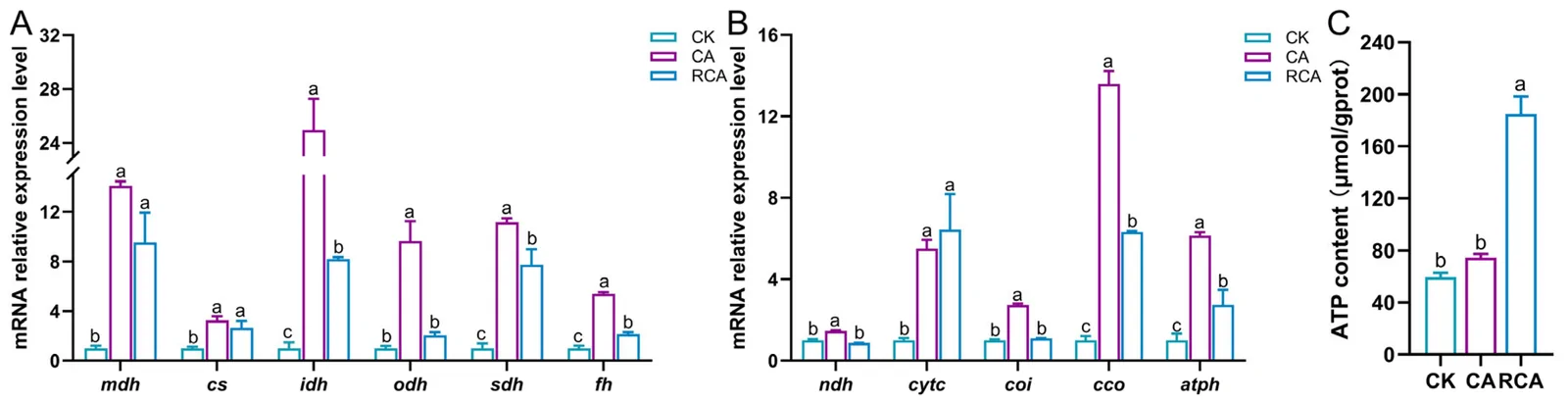
Tricarboxylic acid (TCA)-cycle and electron-transport-chain (ETC) indices in the hepatopancreas during CA stress and recovery. (**A**) TCA cycle-related genes. (**B**) ETC-related genes. (**C**) Adenosine triphosphate (ATP) content. *n* = 3 tanks per group, with six shrimp sampled per tank for biochemical assays and three shrimp sampled per tank for gene expression analysis. Groups labeled with different letters represent statistically significant differences (*p* < 0.05). ATP synthase (*atph*); citrate synthase (*cs*); cytochrome c oxidase (*cco*); cytochrome oxidase I (*coi*); cytochrome c (*cytc*); fumarate hydratase (*fh*); isocitrate dehydrogenase (*idh*); malate dehydrogenase (*mdh*); NADH dehydrogenase (*ndh*); 2-oxoglutarate dehydrogenase (*odh*); succinate dehydrogenase (*sdh*).

After CA stress, the CA group exhibited significantly increased expression of malate dehydrogenase (*mdh*), citrate synthase (*cs*), isocitrate dehydrogenase (*idh*), 2-oxoglutarate dehydrogenase (*odh*), succinate dehydrogenase (*sdh*), and fumarate hydratase (*fh*) relative to the CK group (*p* < 0.05) (Figure 6A).

During recovery, *mdh* and *cs* expression showed a slight decrease relative to the CA group (*p* > 0.05), but remained above the CK baseline (*p* < 0.05). In contrast, *idh*, *sdh*, and *fh* expression declined significantly relative to the CA group (*p* < 0.05), although their levels were still higher than those observed in the CK group (*p* < 0.05). The expression of *odh* also decreased significantly in the RCA group (*p* < 0.05), with no significant difference from the CK group.

#### 3.8.4. Electron Transport Chain (ETC)

CA exposure significantly increased NADH dehydrogenase (*ndh*), cytochrome oxidase I (*coi*), cytochrome C (*cytc*), cytochrome c oxidase (*cco*), and ATP synthase (*atph*) transcripts (*p* < 0.05) relative to the CK group, while ATP content rose slightly (*p* > 0.05) (Figure 6B,C).

Following recovery, relative to the CA group, *ndh* and *coi* expression in the RCA group was significantly reduced (*p* < 0.05), and returned to CK levels (*p* > 0.05). Similarly, *cco* and *atph* expression declined significantly in the RCA group, while remaining significantly higher than CK group (*p* < 0.05). In contrast, *cytc* expression showed no significant change in the RCA group (*p* > 0.05), but exceeded CK values (*p* < 0.05). ATP content increased significantly in the RCA group and exceeded CK values (*p* < 0.05).

### 3.9. Changes of the Metabolite Patterns of the Hepatopancreas

#### 3.9.1. Identification of Differential Metabolites

**Figure 7:**
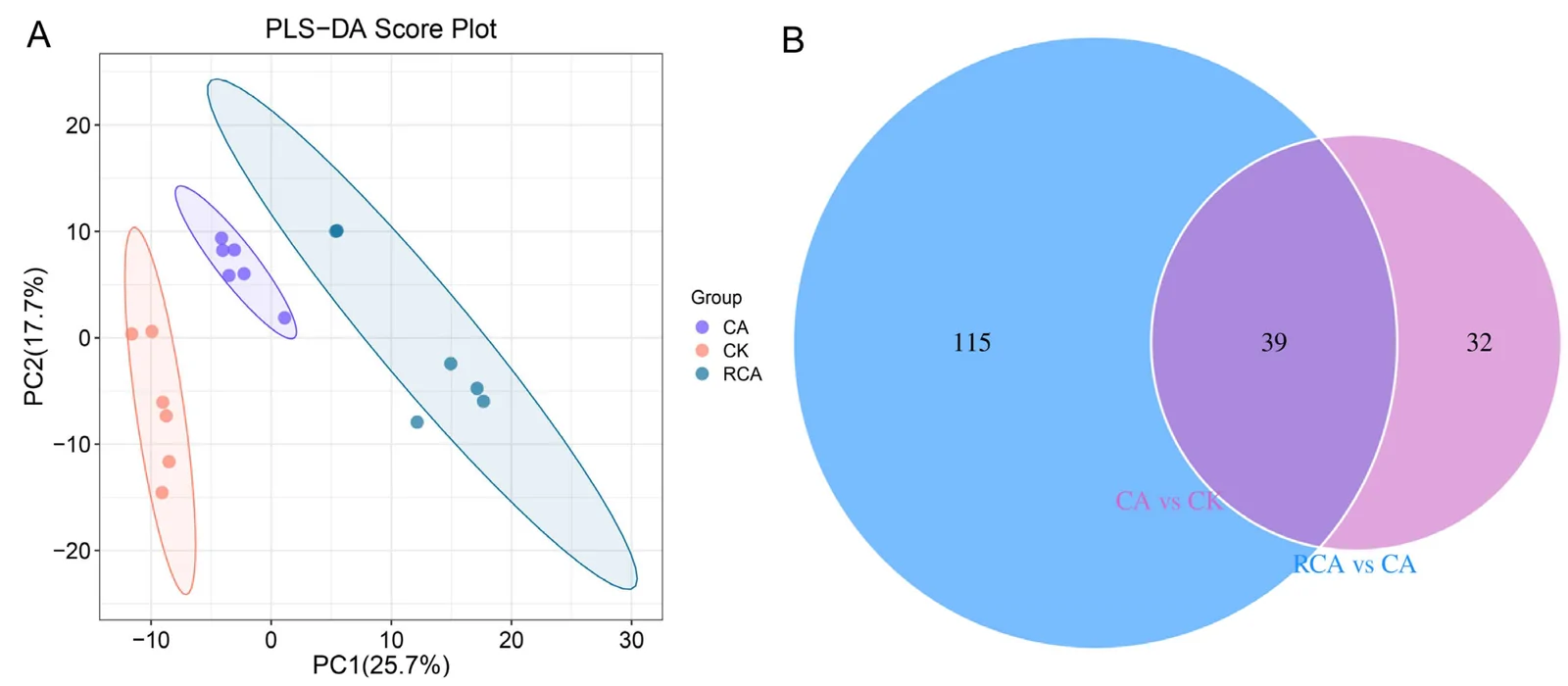
Alterations in the hepatopancreatic metabolite profiles of *L. vannamei* after CA stress and recovery. (**A**) Partial least squares discriminant analysis (PLS-DA) score plot. (**B**) Venn diagram of differential metabolites. *n* = 3 tanks per group, with six shrimp sampled per tank.

LC–MS/MS was used to characterize hepatopancreatic metabolic profiles. PLS-DA clearly separated the three groups (Figure 7A). Metabolomic profiling identified 380 metabolites, including 43 increased and 28 decreased metabolites in the CA group relative to the CK group. Compared with CA group, the RCA group had 27 up-regulated and 127 down-regulated metabolites. 39 differential metabolites were shared between the two comparisons, and the CA-versus-CK comparison contained more unique metabolites than RCA versus CA (Figure 7B).

#### 3.9.2. Pathway Analysis of Differential Metabolites

**Figure 8:**
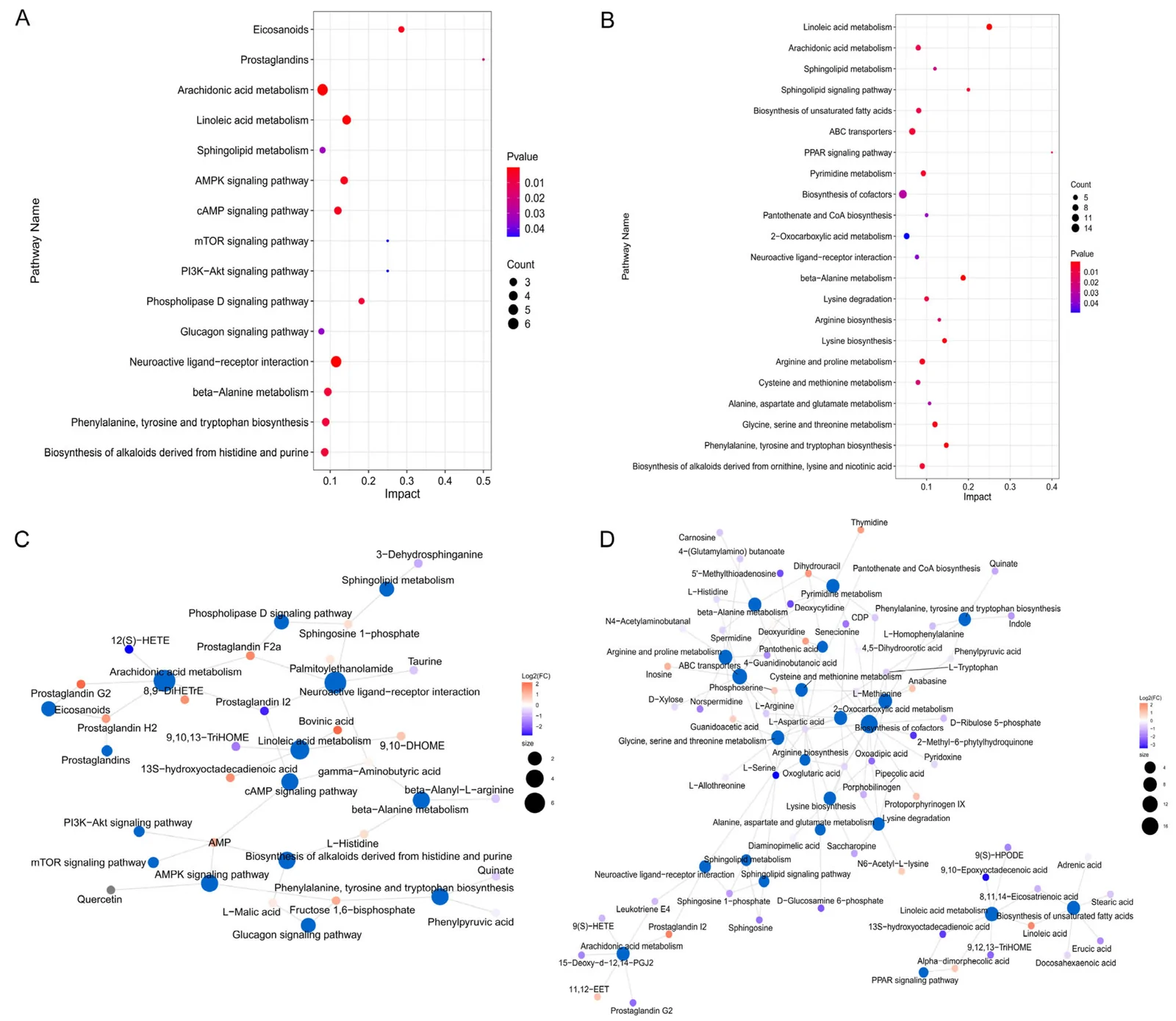
Pathway enrichment and metabolite–pathway networks in the *L. vannamei* hepatopancreas during CA exposure and recovery. (**A**) Enriched pathways for CA versus CK. (**B**) Enriched pathways for RCA versus CA. (**C**) Correlation network for CA versus CK. (**D**) Correlation network for RCA versus CA. *n* = 3 tanks per group, with six shrimp sampled per tank. Cyclic adenosine monophosphate (cAMP); peroxisome proliferator-activated receptor (PPAR); ATP-binding cassette (ABC); γ-aminobutyric acid (GABA); 8,9-dihydroxyeicosatrienoic acid (8,9-DiHETrE); 9,10-dihydroxyoctadecenoic acid (9,10-DHOME); 9,10,13-trihydroxyoctadecenoic acid (9,10,13-TriHOME); 12(S)-hydroxyeicosatetraenoic acid (12(S)-HETE).

Differential metabolites were subjected to pathway enrichment analysis. Significant changes were observed in 33 of the 71 pathways between the CA and CK groups. Compared with the CA group, of 154 pathways in the RCA group, 40 pathways showed significant alterations. Among these, the pathways “*β*-alanine metabolism,” “phenylalanine, tyrosine, and tryptophan biosynthesis,” “arachidonic acid metabolism,” “linoleic acid metabolism,” and “sphingolipid metabolism” showed significant changes in both comparisons. Additionally, significant enrichment restricted to the CA-versus-CK comparison was observed for the “AMPK signaling pathway,” “cyclic adenosine monophosphate (cAMP) signaling pathway,” and “glucagon signaling pathway” (Figure 8A). Conversely, the “glycine, serine, and threonine metabolism,” “alanine, aspartate, and glutamate metabolism,” “cysteine and methionine metabolism,” “arginine biosynthesis,” “lysine biosynthesis,” “biosynthesis of ornithine, lysine, and nicotinoid alkaloids,” “lysine degradation,” “biosynthesis of unsaturated fatty acids,” “sphingolipid signaling pathway,” “peroxisome proliferator-activated receptor (PPAR) signaling pathway,” and “ATP-binding cassette (ABC) transporters” pathways showed significant enrichment specifically in the RCA group (Figure 8B).

Interactions among the significantly enriched pathways identified above were further examined. In the CA group, “arachidonic acid metabolism” and “cAMP signaling pathway” were interrelated through the metabolite prostaglandin I2; “glucagon signaling pathway”, “phenylalanine, tyrosine, and tryptophan biosynthesis”, and “AMPK signaling pathway” were interrelated through the metabolite fructose 1,6-bisphosphate; “biosynthesis of histidine and purine-derived alkaloids”, “AMPK signaling pathway”, and “cAMP signaling pathway” were interrelated through the metabolite AMP; “*β*-alanine metabolism” and “cAMP signaling pathway” were interrelated through the metabolite γ-aminobutyric acid (GABA) (Figure 8C). In the RCA group, “linoleic acid metabolism” and “unsaturated fatty acid biosynthesis” were interrelated through the metabolites linoleic acid and 8,11,14-eicosatrienoic acid; “PPAR signaling pathway” and “linoleic acid metabolism” were interrelated through the metabolites 13(S)-hydroxyoctadecadienoic acid (13(S)-HODE) and α-diphosphate; “glycine, serine and threonine metabolism” and “phenylalanine, tyrosine and tryptophan biosynthesis” were interrelated through the metabolite L-tryptophan; “glycine, serine and threonine metabolism”, “cysteine and methionine metabolism”, “alanine, aspartate, and glutamate metabolism”, “ornithine, lysine and nicotinic acid alkaloid biosynthesis”, “arginine biosynthesis”, and “ABC transporters” were interrelated through the metabolite L-aspartic acid; “sphingolipid metabolism”, “ABC transporters”, “glycine, serine and threonine metabolism”, and “sphingolipid signaling pathway” were interrelated through the metabolite L-serine; “ABC transporters” and “*β*-alanine metabolism” were interrelated through the metabolite L-histidine (Figure 8D).

#### 3.9.3. The Variation Characteristics of Potential Metabolite Candidates

**Figure 9:**
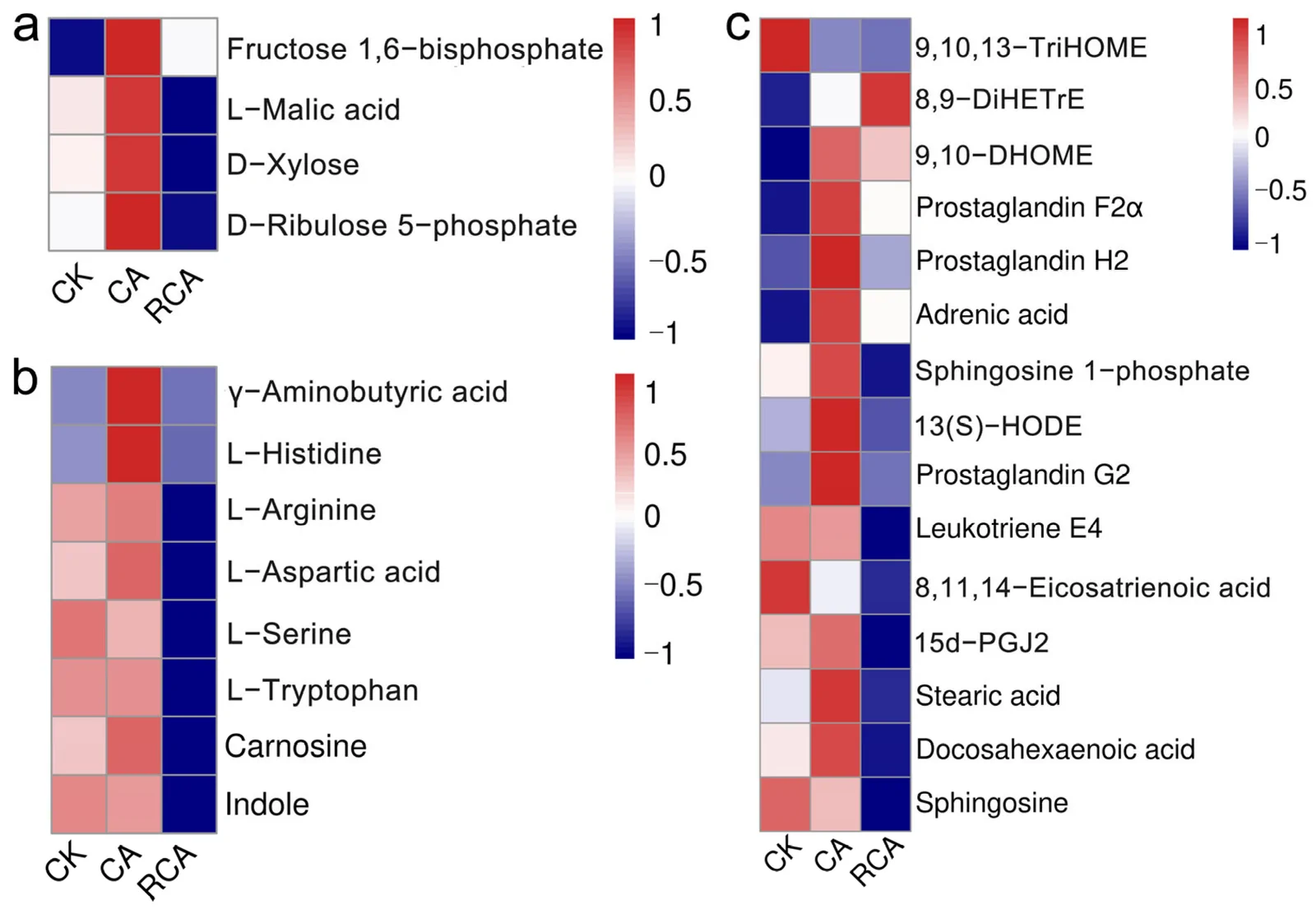
Candidate metabolites associated with the CA response in the *L. vannamei* hepatopancreas. (**a**) Carbohydrate and derivatives. (**b**) Amino acid and derivatives. (**c**) Lipid and derivatives. *n* = 3 tanks per group, with six shrimp sampled per tank. 13(S)-hydroxyoctadecadienoic acid (13(S)-HODE).

The change characteristics of functional differential metabolites related to stress response were further analyzed (Figure 9, Table S2). Among the four carbohydrates and their derivatives, the CA group exhibited the elevated levels of fructose 1,6-bisphosphate, L-malic acid, and D-xylose relative to the CK group. After stress removal, D-xylose returned to CK-like values, whereas 5-phosphate-D-ribulose remained below CK.

Among the eight amino acids and their derivatives, GABA and L-histidine showed increased abundance following CA exposure. After recovery, GABA did not differ from the CA group and returned to CK levels. L-tryptophan and L-histidine concentrations returned to CK levels, whereas L-arginine, L-aspartic acid, L-serine, carnosine, and indole showed significant decreases during recovery.

Among the 16 detected lipids and derivatives, relative to the CK group, 8,9-dihydroxyeicosatrienoic acid (8,9-DiHETrE), 9,10-dihydroxyoctadecenoic acid (9,10-DHOME), prostaglandin F2α, prostaglandin H2, prostaglandin G2, adrenic acid, sphingosine 1-phosphate, and 13(S)-HODE were elevated in the CA group, whereas 9,10,13-trihydroxyoctadecenoic acid (9,10,13-TriHOME) and 12(S)-hydroxyeicosatetraenoic acid (12(S)-HETE) were reduced. During recovery, the RCA group showed no significant changes in 9,10-DHOME, prostaglandin F2α, or prostaglandin H2 compared with the CA group, and these metabolites returned to CK levels. Conversely, adrenic acid, prostaglandin G2, 15-deoxy-d-12,14-prostaglandin J2, 8,11,14-eicosatrienoic acid, stearic acid, and docosahexaenoic acid decreased to values comparable with CK. Additionally, sphingosine 1-phosphate, 13(S)-HODE, leukotriene E4, and sphingosine also decreased during recovery and ended below CK levels. Both 9,10,13-TriHOME and 12(S)-HETE exhibited slight decreases in the RCA group, also falling below the CK group levels. On the other hand, 8,9-DiHETrE showed a slight increase in the RCA group, surpassing the levels found in the CK group.

## 4. Discussion

Shrimp aquaculture in saline–alkali areas has considerable potential and represents an important approach to the comprehensive utilization of saline–alkali resources. However, high CA is a major constraint on shrimp survival and physiological stability in saline–alkaline culture systems [5]. In the present study, CA stress caused hepatic tubule atrophy and basement membrane separation in the hepatopancreas of *L. vannamei*, consistent with previous reports of CA-induced hepatopancreatic injury [5,10], indicating compromised tissue integrity with potential consequences for hepatopancreatic function. During recovery, the lesions partially regressed after stress removal but remained evident relative to the CK group, indicating incomplete structural recovery. Thus, this study integrated multiple biological levels to characterize the hepatopancreatic responses of *L. vannamei* to CA stress.

### 4.1. Physiological Responses of the Hepatopancreas Under CA Stress and Recovery

Environmental stress promotes ROS accumulation and triggers oxidative stress in aquatic animals [18,19,20,21,22]. ROMO1/Nrf2-mediated antioxidant responses and HSP70-associated protein protection are important components of cellular stress defense [23,24,25,26]. Nrf2-regulated SOD and GPx cooperate to scavenge excess ROS [27]. In this study, the CA-induced increases in *romo1*, *nrf2*, *gpx*, and *hsp70* suggested activation of antioxidant and protein-protective defenses in the hepatopancreas. During recovery, *romo1*, *nrf2*, and *gpx* remained higher than in the control group, whereas *sod* and *hsp70* returned to control levels, suggesting incomplete restoration of redox homeostasis.

Excessive ROS accumulation can disrupt calcium homeostasis and the protein-folding capacity of the ER, thereby contributing to ER stress [28]. The Bip-IRE1-XBP1 axis is an important component of the ER stress response [29]. In this study, the elevated expression of *bip*, *ire1*, and *xbp1* after CA stress suggested the involvement of the unfolded protein response and ER-stress-related signaling. During recovery, the expression of these genes generally declined, suggesting partial relief of ER stress. Nevertheless, *ire1* and *xbp1* gene expression remained above control levels, implying incomplete restoration of ER homeostasis and possible mild residual stress to maintain proper protein folding.

To handle environmental stress, shrimp primarily utilize non-specific immune factors [30]. Key molecules in their immune system include ALF, proPO, Crus, Pen-3, and Lys [31,32,33,34]. The concurrent rise in *alf*, *crus*, *pen-3*, *lys*, and *propo* suggested that hepatopancreatic immune defenses were mobilized by CA stress. During recovery, *alf*, *crus*, *pen-3*, and *propo* gene expression returned to the control levels, whereas *lys* gene remained elevated. This implies that although immune homeostasis is approaching normalization, it is not yet fully achieved. The sustained high expression of *lys* might contribute to subsequent tissue repair.

CA stress can induce changes in apoptosis-related signaling, in which Casp-9 and Casp-3 function as key regulators [35,36]. In this study, CA exposure increased *casp-3* and *casp-9* expression, suggesting enhanced pro-apoptotic signaling characteristic of the intrinsic apoptotic pathway. During recovery, *casp-3* gene expression remained elevated, while *casp-9* gene expression decreased significantly but remained higher than the control level, suggesting that apoptotic signaling was attenuated but had not yet returned to baseline.

Aquatic organisms activate their detoxification systems in response to environmental stress [1]. CYP450 and GST are involved in phase I and phase II detoxification, respectively [37,38]. In this study, the upregulation of the *cyp450* gene following CA stress indicated that the hepatopancreas of *L. vannamei* responded by enhancing its phase I detoxification capacity. During recovery, despite a decrease in *cyp450* expression, its level remained above the control level, suggesting sustained transcriptional regulation of phase I detoxification during recovery.

CA stress can influence osmoregulation by altering acid–base and ionic homeostasis in aquatic animals [39]. CA, NKA, and VATP are key regulators of acid–base balance and active ion transport [40,41,42]. In this study, upregulation of *ca*, *nka-α*, *nka-β*, and *vatp* implied activation of ion transport induced by CA stress. During recovery, *ca*, *nka-β*, and *vatp* remained upregulated relative to controls, suggesting that the osmoregulatory function had not fully normalized.

Membrane transport proteins can further coordinate ion exchange and transmembrane water transport to maintain osmotic balance. NHE, AQP, CLC, and CCP are involved in these processes [43,44,45]. In this study, higher *nhe* and *aqp* expression under CA stress suggested increased ion exchange and water movement to support osmotic balance in the hepatopancreas. During recovery, *nhe*, *aqp*, and *ccp* stayed above control levels, while *clc* and *tip4* returned to baseline, implying that osmotic regulation had not yet fully recovered.

### 4.2. Energy Metabolism Responses of the Hepatopancreas Under CA Stress and Recovery

Environmental stress increases energy demand, prompting metabolic adjustments to maintain physiological homeostasis [1]. In this study, CA stress decreased PYR content and increased the expression of glycolysis-related genes (*hk*, *pk*, *pdh*, and *ldh*), suggesting enhanced regulation of glycolysis to meet the increased energy demand. During recovery, LAC returned to the control level, whereas GLU and PYR remained lower than the control levels and glycolysis-related genes (*hk*, *pk*, *pdh*, and *ldh*) remained above control values. These findings suggested that glycolytic metabolism might have been partially restored but had not yet fully returned to the basal state.

Lipids represent a major form of energy storage in organisms [46]. AMPK, TG, SREBP, ACC, and FAS are important indicators of lipid metabolism, with distinct roles in lipid catabolism and energy production, lipid energy storage, and fatty acid biosynthesis [47,48,49]. In this study, CA stress reduced TG content and increased the expression of *ampk*, *srebp*, *acc*, and *fas*, suggesting substantial adjustment of lipid metabolism and mobilization of lipid reserves in response to increased energy demand. During recovery, TG content remained below the control level and *ampk* and *acc* expression remained elevated, whereas *srebp* and *fas* returned to control levels. These changes suggested that lipid synthesis was gradually restored during recovery, while lipid reserves remained insufficiently replenished and lipid utilization continued to support energy demands.

The TCA cycle is a central energy-producing pathway whose activity depends on enzymes including CS, IDH, ODH, SDH, FH, and MDH [50,51,52]. In this study, the coordinated increase in *mdh*, *cs*, *idh*, *odh*, *sdh*, and *fh* after CA stress suggested increased TCA-cycle regulation to support elevated energy requirements. During recovery, most of these genes remained above control levels, whereas *odh* returned to baseline, suggesting that TCA cycle-related metabolic regulation had only partially recovered.

The ETC establishes a proton gradient across the mitochondrial inner membrane, thereby driving ATP synthase and supporting cellular ATP production [1]. NDH, CytC, COI, CCO, and Atph are essential components of ETC that contribute to ATP synthesis and maintain the mitochondrial electrochemical gradient [53,54,55,56]. In this study, the rise in *cco*, *cytc*, *coi*, *ndh*, and *atph* after CA stress suggested enhanced transcriptional regulation of mitochondrial electron transport and ATP-generating capacity. During recovery, *ndh* and *coi* expression normalized, whereas *cytc*, *cco*, and *atph* remained elevated, along with ATP content. These results suggested that mitochondrial energy metabolism remained actively regulated during the recovery period and had not yet fully returned to the control state.

### 4.3. Metabolomic Responses of the Hepatopancreas Under CA Stress and Recovery

CA stress is known to affect the metabolic function of aquatic animals [8]. CA stress and subsequent recovery altered the metabolite profiles of the *L. vannamei* hepatopancreas. Enrichment analysis showed that “*β*-alanine metabolism”, “phenylalanine, tyrosine and tryptophan biosynthesis”, “arachidonic acid metabolism”, “linoleic acid metabolism” and “sphingolipid metabolism” were all significantly altered during both stress and recovery phases, suggesting prominent remodeling of amino acid and lipid metabolism under CA stress. In addition, “AMPK signaling pathway”, “cAMP signaling pathway”, and “glucagon signaling pathway” were significantly enriched only in the CA stress group, suggesting that CA stress might cause hepatopancreatic metabolic disorder by interfering with energy metabolism and signal transduction. However, “PPAR signaling pathway” and several amino acid- and lipid-related pathways were only significantly enriched in the RCA group, suggesting a metabolic shift from stress-associated energy mobilization toward nutrient reutilization and energy reserve restoration during recovery.

The potential metabolic markers of CA stress were further screened. Fructose 1,6-bisphosphate and malic acid are intermediates of glycolysis and the TCA cycle, respectively [57,58,59], whereas 5-phosphate-D-ribulose is involved in pentose metabolism [60]. In this study, the levels of fructose 1,6-bisphosphate, L-malic acid, and D-xylose increased under CA stress, suggesting alterations in carbohydrate metabolism that might be related to increased energy demand. During recovery, D-xylose returned to control levels, while 5-phosphate-D-ribulose dropped below control levels, suggesting that carbohydrate metabolic homeostasis had not yet fully normalized.

Amino acid metabolism is the key target affected by CA stress. Amino acids and their derivatives are closely associated with energy metabolism, immune regulation, and antioxidant defense [61,62,63,64,65,66]. In the present study, the elevation of L-histidine and GABA during CA stress, followed by normalization during recovery, suggested possible roles in stress adaptation. In contrast, carnosine, indole, L-serine, L-arginine, L-aspartic acid, and L-tryptophan showed no significant changes under CA stress, but decreased below control levels during recovery. The post-stress decline of these metabolites might reflect their increased utilization or redistribution to support immune and energy-related processes during recovery, suggesting that the restoration of metabolic homeostasis was still ongoing after the removal of CA stress.

Lipid metabolic pathways are important components of the physiological response of aquatic animals to environmental challenges. Linoleic acid metabolism is crucial for antioxidant responses [67]. In this study, CA stress was accompanied by elevated 13(S)-hydroxyoctadecadienoic acid and reduced 9,10,13-trihydroxyoctadecenoic acid, suggesting remodeling of linoleic acid-related metabolism that may be associated with antioxidant regulation. During recovery, both metabolites remained below control levels, demonstrating that linoleic acid metabolism had not fully recovered. Arachidonic acid and its derivatives serve as key immunomodulatory molecules [68]. In the present study, CA stress elevated prostaglandin F2α, prostaglandin H2, and prostaglandin G2, suggesting that stress might impact immune homeostasis in the *L. vannamei* hepatopancreas by arachidonic acid metabolism. In the recovery stage, prostaglandin F2α, prostaglandin H2 and 9,10-dihydroxy octadecenoic acid returned to the control levels, while leukotriene E4 and 15-deoxy-d-12,14-prostaglandin J2 remained at a low level, suggesting that arachidonic acid metabolism was not fully restored and immune regulation and inflammatory responses persisted. Unsaturated fatty acids contribute to cell membrane fluidity, support energy metabolism, and have anti-inflammatory and antioxidant effects [69]. In the present study, adrenic acid and sphingosine 1-phosphate were increased under CA stress, suggesting that unsaturated fatty acids might participate in cellular stress responses and protective mechanisms. During recovery, adrenic acid, stearic acid and docosahexaenoic acid recovered to the control levels, while sphingosine 1-phosphate was decreased below the control level, suggesting incomplete recovery of unsaturated fatty acids metabolism and that a longer period might be required to re-establish cell membrane stability and functional balance.

Nevertheless, some limitations of the present study need to be considered. For example, the involvement of ROS in oxidative stress was assessed primarily based on changes in selected molecular indicators, while histopathological alterations were mainly evaluated qualitatively. Future studies incorporating ROS-related functional validation, quantitative lesion scoring, and blinded histopathological assessment would help further clarify the role of ROS in CA-induced physiological disturbances and provide a more objective evaluation of hepatopancreatic injury. In addition, this study adopted a single CA concentration and single exposure and recovery durations. Future studies incorporating multiple CA concentrations and additional sampling points could further elucidate the responses and recovery processes of *L. vannamei* under CA stress.

## 5. Conclusions

**Figure 10:**
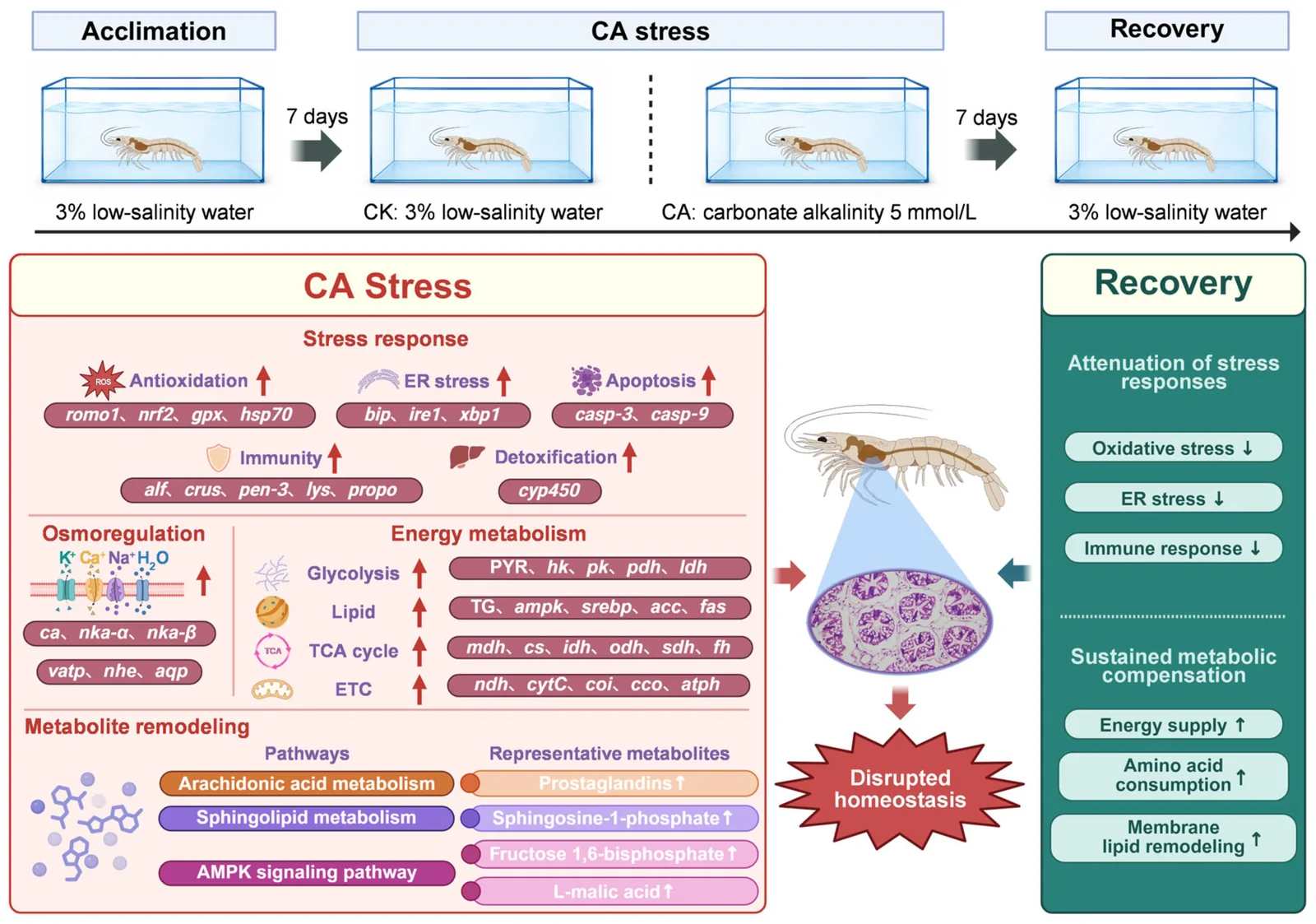
Derivation of toxicological response mechanisms of the hepatopancreas in *L. vannamei* during CA exposure and recovery. ↑ and ↓ indicate increases and decreases, respectively, during CA stress or recovery.

CA stress resulted in hepatopancreatic structural damage and triggered oxidative- and ER-stress responses and enhanced apoptotic signaling. The organism exhibited coordinated responses to stress, characterized by enhanced antioxidant defenses, immune responses, and phase I detoxification-related processes, along with the regulation of ion and water transport and energy metabolism through glycolysis, lipid metabolism, the TCA cycle, and the ETC. After stress removal, some physiological indices partially recovered, but the overall function was not fully restored. Additionally, CA stress also disrupted hepatopancreatic metabolic regulation. The glucagon signaling and the metabolism of *β*-alanine, arachidonic acid, and linoleic acid functioned during the stress stage, whereas ABC transporters and the metabolism of *β*-alanine, arachidonic acid, linoleic acid, and unsaturated fatty acids functioned during the recovery stage (Figure 10). Additional functional metabolites associated with stress responses were further identified, potentially guiding the development of anti-stress functional substances.

## Data Availability

Data will be made available upon request.

## Supplementary Materials

The following supporting information can be downloaded at: https://www.mdpi.com/article/10.3390/antiox15091052/s1, Table S1: Primer sequences used in this study. Table S2: Changes of differential metabolite biomarkers in the hepatopancreas of *Litopenaeus vannamei* under carbonate alkalinity stress and recovery. Materials and methods.
