# Supplementary material for: Physiological Responses in the Hepatopancreas of Litopenaeus vannamei to Carbonate Alkalinity Stress and Subsequent Recovery: Integration of Antioxidant, Immune, and Metabolic Profiles
Source: Antioxidants (Basel). 2026 Aug 23;15(9):1052. doi: 10.3390/antiox15091052 (PMC13603219; doi:10.3390/antiox15091052)
Supplement: Supplementary file 1 [file antioxidants-15-01052-s001.zip › antioxidants-4474259-supplementary.pdf]

Supplementary materials

Table S1. Primer sequences used in this study.

Gene name	Forward primer (5′-3′)	Reverse primer (5′-3′)
romo1	GCACCGTGGAGTAGGAGAACAAC	TTGCCATGCCGACTGAGAAACC
nrf2	GATGAGAAGCGAGCCAGAGCG	GCCGTCGGATGTCTCGGATAA
sod	GACACGACCATTAGCCTGTACGAC	CAGCGTTGCCAGTAGCGAGTG
gpx	TCTGAGCGGCGAGATGGTGTC	CTGGTGGAAGTCCCTGGTGGTC
bip	CCAGCACGACATCCAGTTCTTCC	CCTCCGCAGCAAACACCTTCTC
ire1	GGTACATTAGGTTCTCGTCCGTCAC	AATTCCTCTGGTGTTGCCTTAGCC
xbp1	CGCCTGCTGAGGATGACCTTATTAC	GCCTACTGGTGATGTGTCCTTAACG
casp-3	AGACGGACAGCATACAGGAGGAC	CTCGGCCAAGAAGTGGATGAAGAC
casp-9	ATGGCTCGTGGTTCATTCAG	CATCAGGGTTGAGACAATACAGG
alf	GGTGTTCCTGGTGGCACTCT	AGCTCCGTCTCCTCGTTCCT
crus	CCACGAACCAGAGACACCTG	CGAGGCCAGCACACTTGTAG
lys	TCGAGTCGTCCTTCAACACG	TGCAGACGTTCTTGCCGTAG
propo	CAATGACCAGCAGCGTCTTC	CACGGAAGGAGGCGTATCAT
cyp450	CAACGACACCATCAGGAGCAGAC	CAGCAGCAGGTCCAAGAAGGC
gst	ACGAACACTACGAACAGAAGGATGC	GCCAGGAAGTCGATGTAGGTTAGC
nka-a	TGAAATCGTGTTTGCCCGTACCTC	ACCATCACCAGTTACAGCCACAATG
nka-β	GAACCCAGCCGACGAAGAATACG	CAGCAACAATAGGTGGCAGGTAGC
vatp	GCCAAGTATGAGCACCTAGCAGAC	TCGTCCACCACCTTACTGAAGAGAG
ca	CCTATTCTGGCTCCCTCACTACCC	GTTCATCCTCTGGGCAACACTCG
nhe	CACCGCCATCCTCACCAAGTTC	ACAGAAGAGCACAGCCACAATACC
aqp	GCAGCCATCTTGAAGGGAGTGAC	ACGAGGACGAAGGTGATGAGGAG
tip4	GTCTCTCTGAACCCTTGCCAAACTC	GTTGCCTATGATTACCTGCGTCCTC
clc	GCCGATAGGAGGTGTGTTGTTCAG	CGAAGAAGCCACGCCAGTAGTTC
ccp	CCCTGAGACCTCTGTTTGCTGTG	TGGCAGTGTTCTTCGCATGTTCC
pdh	TCAGCCTCAACCAACTACTACACTC	GCCTCCTTCACACTCAGTACATCC
hk	ACCTGCTGCTGGTTCACGATG	GCTGCTGCCTCCTCCAAGTG
pk	GCCAGACAGTGCCATCTCTACC	TGCCAGCCAGTCACCACAAC
ldh	GATCGGCTCAGGCACCAACC	GCAACATTAACACCAGACCAGACAG
ampk	GTAGTCCACGATCACCACTCAATGC	CCACCTGCTCCTCCTACTCCATC
srebp	AGATGGCTGAGATGTTGGTAATGGC	CCCTTGTGGCTCTTCCTCTTTGC
acc	CGGCAGACAACATCCATACCACAG	GCAACCAGCGAGAGCAGTAACC
fas	TGCTACTGTGCCTGTTGTGTATGC	CCACCAGAACCTGCGTGAATGAG
cs	GCTCGGTTCCATCCATCCTCTG	ACGCCTTCTGTTGGTGTCCTAAG
idh	ACGGAGACCAATACAAGGCTACTG	TGAGTGTGCGAAGGAACGGATAG
odh	GCAAGGCATAATCAGGGCATATCAG	CATAGGAGCGGACTACTGTTCTGG
sdh	TTCCTGGCACTCACTATGACTGTTC	GATGAAGTAGCAGAGACCTCCCAAG
fh	TTGTTCCTCTTCTCGCCTGGTTAC	TCTGACTCCATCTTTGCCACTCTTC
mdh	CTCTTCCACCCAGTTCCCAGATG	GACAACTTACGAGCGGCAATGAC

ndh	CTTCTTGGTTCGGTGCTTGAATGG	AGCAGCCTCTGAAGAGTATTGGTTG
coi	CCCAGATATAGCCTTCCCTCGAATG	GCGTGAGCAATACTGGCAGATAAAG
cco	ATGCCAGGTGTCCGCTTCAAG	AAGGGTCAACTTGTTCCAGTCTCC
cytc	TCGACGTGTACCTGACCAACCC	TTCGCCTGGCTTCCTCTTCCTC
atph	CACCATCATCAACCAGAAGCGATTC	GGAGCAGCATCAGAGGCAGTG
β-actin	TCGCTCCCTCCACCATGAAGATC	CTCCTGCTTGCTGATCCACATCTG

Table S2. Changes of differential metabolite biomarkers in the hepatopancreas of *Litopenaeus vannamei* under carbonate alkalinity stress and recovery.

Metabolites	Log2FC		Categories
	CA vs CK	RCA vs CA	
Fructose 1,6-bisphosphate	1.22	/	Carbohydrate and derivatives
L-Malic acid	0.32	/	Carbohydrate and derivatives
D-Xylose	0.22	-0.56	Carbohydrate and derivatives
D-Ribulose 5-phosphate	/	-0.99	Carbohydrate and derivatives
γ-Aminobutyric acid	0.18	/	Amino acid and derivatives
L-Histidine	0.49	-0.56	Amino acid and derivatives
L-Arginine	/	-0.78	Amino acid and derivatives
L-Aspartic acid	/	-0.61	Amino acid and derivatives
L-Serine	/	-3.42	Amino acid and derivatives
L-Tryptophan	/	-0.75	Amino acid and derivatives
Carnosine	/	-0.86	Amino acid and derivatives
Indole	/	-1.53	Amino acid and derivatives
8,9-DiHETrE	1.61	/	Lipid and derivatives
9,10-DHOME	0.85	/	Lipid and derivatives
9,10,13-TriHOME	-1.65	/	Lipid and derivatives
Adrenic acid	0.68	-0.28	Lipid and derivatives
Sphingosine 1-phosphate	0.48	-1.46	Lipid and derivatives
13(S)-HODE	1.63	-2.91	Lipid and derivatives
Prostaglandin G2	2.12	-2.25	Lipid and derivatives
Prostaglandin F2α	1.71	/	Lipid and derivatives
Prostaglandin H2	1.49	/	Lipid and derivatives
Leukotriene E4	/	-1.06	Lipid and derivatives
8,11,14-Eicosatrienoic acid	/	-1.15	Lipid and derivatives
11,12-EET	/	1.06	Lipid and derivatives
15-deoxy-d-12,14-prostaglandin J2	/	-1.85	Lipid and derivatives
Stearic acid	/	-0.86	Lipid and derivatives
Docosahexaenoic acid	/	-0.61	Lipid and derivatives
Sphingosine	/	-2.07	Lipid and derivatives

2. Materials and methods

2.1. Shrimp and culture conditions

The healthy *L. vannamei* were sourced from an indoor industrialized aquaculture pond of the Shenzhen Base of South China Sea Fisheries Research Institute, Chinese Academy of Fishery Sciences (Shenzhen, China), the average weight of each shrimp was 9.6 ± 0.4 g. Before exposure, shrimp were acclimated for 7 days in 300-L tanks maintained at 25 ± 0.5 °C, salinity 3‰, and pH 8.2 ± 0.2, with continuous aeration and complete daily wa-ter exchange. The tank was continuously aerated throughout the 24 h period. The total volume of water was completely renewed daily. Mixed feed for *L. vannamei* was supplied at 5% of shrimp weight, with feed amounts adjusted as needed based on feeding behavior. Uneaten feed and feces were promptly removed to maintain water quality.

2.2. Carbonate alkalinity (CA) stress test and sample collection

After a week of acclimatization, the shrimp were randomly divided into two groups: the CA stress group and the control (CK) group. Three replicate tanks (98.0 cm inner diameter, 100.0 cm depth, 300 L effective water volume) were assigned to each group, with 50 shrimp per tank. Based on previous studies and the CA ranges observed in saline-alkaline aquaculture ponds [1–4], 5 mmol/L CA was selected as the exposure concentration for the present study. The CK group was maintained in low-salinity water (salinity 3‰), while the CA group had sodium bicarbonate added to the low-salinity water to achieve a concentration of 5 mmol/L CA. A 100% water exchange was performed daily in each tank using pre-prepared experimental water, and other culture conditions remained unchanged. CA was determined by acid–base titration. According to previous studies, a 7-day stress duration was

adopted to investigate the response of *L. vannamei* to CA stress. A 7-day recovery period has also been shown to reflect short-term physiological recovery patterns following stress removal [2,5,6]. After 7 days of CA stress, a new RCA group was created, which involved replacing the culture water in the CA group with normal 3‰ low-salinity water. Subsequently, 15 shrimp from each CA tank were transferred to normal culture conditions for a further 7 days.

The hepatopancreas of the shrimp were sampled on the 7th day of stress exposure and the 7th day of subsequent recovery for corresponding analyses. Six shrimp were randomly sampled from each tank, and their hepatopancreas were divided into two halves: one half was used for biochemical analysis, and the other half for metabolomic analysis, all samples were stored at -80 °C. Another three shrimp were sampled from each tank, and their hepatopancreas were collected and pooled in RNA preservation solution (NCM Biotech, Suzhou, China) for gene expression analysis. Additionally, the hepatopancreas of three shrimp per tank were collected and fixed in 4% paraformaldehyde for histological examination.

2.3. Histomorphological analysis

The hepatopancreas was fixed in 4% paraformaldehyde 24 h, the hepatopancreas was transferred to 70% ethanol for overnight treatment. Subsequently, samples were dehydrated sequentially with ethanol solutions of increasing concentration (80%, 90%, and 100%), cleared with xylene, and embedded in paraffin. Tissue sections of 4 μm thickness were cut using a microtome. Finally, the sections were stained with hematoxylin and eosin (H&E), and the morphological characteristics of the hepatopancreas were observed and documented under a

microscope.

2.4. Biochemical indexes determination

After thawing the frozen hepatopancreas samples, the cellular fluid was rinsed off with normal saline, blotted dry with filter paper, and weighed. The tissue was then homogenized at low temperature using a tissue homogenizer (TissueLyser II, Qiagen, Hilden, Germany) with a mass ratio of tissue to normal saline of 1:9. We centrifuged the homogenate at 4 °C for 15 min at 1370× *g* (Centrifuge JIDI-21R, Guangzhou, China), collected the supernatant, and stored it at −80 °C for future biochemical measurements. Biochemical parameters, including glucose (GLU), pyruvic acid (PYR), lactic acid (LAC), triglyceride (TG), and adenosine triphosphate (ATP) content, were measured using the same batch of commercial kits from Nanjing Jiancheng Bioengineering Institute (Nanjing, China) according to the manufacturer's instructions.

2.5. Gene expression analysis

Total RNA was first extracted from the hepatopancreas using TRIzol reagent, and residual genomic DNA was removed using RQ1 RNase-Free DNase. The concentration and purity of RNA were measured with a Nanodrop 2000 spectrophotometer (MPBIO, Irvine, CA, USA), and RNA integrity was assessed by 1% agarose gel electrophoresis. Reverse transcription was then performed using the First Strand cDNA Synthesis Kit (Servicebio, Wuhan, China), and the resulting cDNA was stored at -80 °C for subsequent analysis.

The nucleotide sequences of the target genes and the *β-actin* reference gene for *L. vannamei* were retrieved from the National Center for Biotechnology Information (NCBI). Specific primers were designed using Primer Premier 5.0. Primer specificity and amplification

efficiency were validated through amplification plots and melting curve analysis (Table S1). Quantitative real-time PCR (qPCR) was performed on a CG-02 real-time PCR system (Heal Force, Shanghai, China) using SYBR Green Pro Taq HS Premix (Sangon Biotech, Shanghai, China). The 15 μL reaction mixture consisted of 1.0 μL cDNA, 0.6 μL of 10 μmol/L forward primer, 0.6 μL of 10 μmol/L reverse primer, 5.3 μL sterile deionized water, and 7.5 μL of SYBR Green Pro Taq HS Premix (2×). The PCR cycling conditions were initial denaturation at 95 °C for 30 s, followed by 40 cycles of 95 °C for 5 s and 60 °C for 30 s. The relative mRNA level was calculated according to the method of Livak and Schmittgen [7] and presented in the form of multiple changes relative to the CK group.

2.6. The non-targeted metabolomics analysis

Six samples of the hepatopancreas from each group were used for non-targeted metabolomics analysis. The samples were thawed at 4 °C, and the metabolites were extracted using methanol and 2-chlorophenylalanine. From each sample, 20 μL was taken for quality control analysis (QC), while the remaining samples were analyzed using liquid chromatography-mass spectrometry (LC-MS). Liquid chromatography was performed with a Thermo Vanquish system, utilizing an ACQUITY UPLC® HSS T3 1.8 μm column (2.1 × 100 mm, 1.8 μm). Mass spectrometry analysis was conducted using a Thermo Orbitrap Exploris 120 mass spectrometer (Thermo Fisher Scientific, Waltham, USA). The obtained raw metabolomics data were converted to mzXML format (xcms input file format) using Proteowizard software (v3.0.8789). Peak detection, filtering, and alignment were performed using the XCMS (v3.12.2) program in R software. Base peak chromatograms for both positive and negative ion modes were obtained, followed by QC and quality assurance (QA) analysis.

The metabolomics data were standardized using auto-scaling (UV), and multivariate statistical analysis was conducted based on partial least squares discriminant analysis (PLS-DA) using the Ropls package in R. Differential metabolites (DMs) were screened based on $P < 0.05$ and variable importance in projection (VIP) > 1. The false discovery rate (FDR) was controlled using the Benjamini–Hochberg (BH) procedure. Metabolite identification was performed using Metlin, MoNA, and a self-built database from Panomics, with classification based on Kyoto Encyclopedia of Genes and Genomes (KEGG) and Metabolon.inc. Differential metabolite clustering analysis was conducted using the pheatmap program in R (v1.0.12). The KEGG pathways of differential metabolites were analyzed using MetaboAnalyst software, and a metabolic pathway association network was constructed. Based on the annotation results of differential metabolites, further analysis was conducted on the changes in functional metabolites related to the health of the organism.

2.7. Statistical analysis

All the data were presented as mean ± standard error (SE). One-way analysis of variance (ANOVA) was performed using SPSS v27.0, followed by least significant difference (LSD) multiple range tests for post hoc comparisons. Differences were considered statistically significant at $P < 0.05$.

References

1. Duan, Y.F.; Xing, Y.F.; Zhu, X.Y.; Li, H.; Wang, Y.; Nan, Y.X. Integration of transcriptomic and metabolomic reveals carbonate alkalinity stress responses in the hepatopancreas of *Litopenaeus vannamei*. *Aquat. Toxicol.* **2023**, *260*, 106569. https://doi.org/10.1016/j.aquatox.2023.106569.
2. Xiao, M.; Nan, Y.X.; Li, J.T.; Wang, Y.; Zhu, R.J.; Duan, Y.F. Changes in the energy metabolism of the gills of *Litopenaeus vannamei* under carbonate alkalinity stress and recovery conditions. *Front. Mar. Sci.* **2025**, *12*, 1571396. https://doi.org/10.3389/fmars.2025.1571396.
3. Ge, Q.Q.; Li, J.; Wang, J.J.; Li, Z.D.; Li, J.T. Characterization, functional analysis, and expression levels of three carbonic anhydrases in response to pH and saline–alkaline stresses in the ridgetail white prawn *Exopalaemon carinicauda*. *Cell Stress Chaperones* **2019**, *24*, 503–515. https://doi.org/10.1007/s12192-019-00987-z.
4. Song, Z.L.; Li, K.; Li, K. Integrated characterizations of intestinal bacteria and transcriptomics revealed the acute stress response to carbonate alkalinity in white shrimp *Penaeus vannamei*. *Fish Shellfish Immunol.* **2024**, *146*, 109420. https://doi.org/10.1016/j.fsi.2024.109420.
5. Xiao, M.; Nan, Y.X.; Yang, Y.K.; Li, H.; Duan, Y.F. Changes in Physiological Homeostasis in the Gills of *Litopenaeus vannamei* Under Carbonate Alkalinity Stress and Recovery Conditions. *Fishes* **2024**, *9*, 463. https://doi.org/10.3390/fishes9110463.
6. Ríos, V.; Guzmán-Guillén, R.; Moreno, I.M.; Prieto, A.I.; Puerto, M.; Jos, A.; Cameán, A.M. Influence of two depuration periods on the activity and transcription of antioxidant enzymes in tilapia exposed to repeated doses of cylindrospermopsin under laboratory conditions. *Toxins* **2014**, *6*, 1062–1079. https://doi.org/10.3390/toxins6031062.
7. Livak, K.J.; Schmittgen, T.D. Analysis of relative gene expression data using real-time quantitative PCR and the $2^{-\Delta\Delta C_T}$ method. *Methods* **2001**, *25*, 402–408. https://doi.org/10.1006/meth.2001.1262.
